# The Treatment of Pericarditis with Effusion

**Published:** 1899-12-23

**Authors:** 


### THE TREATMENT OF PERICARDITIS WITH EFFUSION.

At a recent meeting of the New York Academy of Medicine, Dr. Lilienthal showed a boy, aged 15 years, who, in the course of an attack of lobar pneumonia, had been attacked with pericarditis. The pneumonia resolved, and for a time the pericardial friction sound was lost, but the precordial dulness increased, the friction returned, the pulse became feeble and irregular, and a month after the first appearance of the friction sound the pericardium was aspirated, 18½ ounces of pus being removed, the pus containing numerous pneumococci. The case was then transferred to the surgical side. Under Eucaine B. (one drachm of a 6 per cent. solution having been used) an incision over two inches long was made from the third to the fifth intercostal space, about half an inch from the sternal border, and joining this a horizontal incision was made along the fifth interspace. About three-quarters of an inch of the fifth intercostal cartilage was removed a quarter of an inch to the outside of the sternum. The hæmorrhage was insignificant. The mediastinal tissues were found to be thickened and adherent, but after passing through an inch of this tissue, under the guidance of an aspirating needle, the pericardium was opened, and a large quantity of pus was evacuated, at least 40 ounces. A portion of the wound was left open for drainage. In about a week daily irrigation with decinormal saline solution was begun. Complete recovery ensued. This is a very good case. There can be but little doubt that when pus is found in the pericardium drainage is the proper course. In regard to this we would refer to a case by Mr. Betham Robinson in the Clinical Society's "Transactions," vol. xxx., and to a paper by Dr. Roberts in the "American Journal of the Medical Sciences" for 1897. Fraenkel also has written to the same effect, recommending resection of a small part of the fifth rib close to the cartilage with free opening of the pericardium, even in a case of rheumatic pericarditis with effusion. The interest of the above case largely hinges on the fact that it was done under local anæsthesia, for these are just the cases in which a general anæsthetic sometimes appears almost out of the question.

